# Currently available medications may not be sufficient for lifelong treatment of HIV

**DOI:** 10.7448/IAS.15.6.18077

**Published:** 2012-11-11

**Authors:** J Jansson, D Wilson, A Carr, K Petoumenos, M Boyd

**Affiliations:** 1 University of New South Wales, Kirby Institute, Sydney, Australia; 2 St Vincent's Hospital, Sydney, Australia

## Abstract

**Purpose of the study:**

Combination antiretroviral therapy (cART) has greatly improved the life expectancy of people living with HIV (PLHIV). A series of cohort studies have predicted near-to-normal life expectancies for PLHIV receiving cART but have not considered the impact of multi-class resistance on long-term survival. Our study aims to project the future life expectancy of PLHIV in a resource-rich setting in the context of the currently available antiretroviral treatments.

**Methods:**

Patient antiretroviral treatment data, including time on each regimen until treatment failure, were sourced from an observational cohort of 3434 predominantly male (94.2%) PLHIV in Australia over the period 1997 to 2010. These data were analyzed in an individual-based mathematical model to calculate the time until exhaustion of all treatment options and the expected impact on HIV-associated mortality. Standardized mortality ratios were used to simulate expected survival before and after treatment exhaustion.

**Summary of results:**

The model estimated that the median time until exhaustion of currently available treatment options is 43.4 years (interquartile range = 31.4 to 58.6 years). However, the model predicts that 10% of PLHIV will use up all currently available cART options after just 22.6 years. The figure shows the survival proportions of males from age 20 years in four mortality scenarios: (1) the general population mortality rate; (2) the mortality rate in PLHIV as currently measured (without considering exhaustion of currently available treatments); (3) mortality rate in PLHIV considering additional mortality due to limited cART options; and (4) mortality rate if no cART is available. PLHIV who start currently available cART regimens at age 20 years are expected to live to a median of 64.7 (95% uncertainty bound (UB) = 61.8 to 69.3) years of age, when adjusting for treatment option exhaustion. This is a substantial improvement on no cART (median survival to 27.6 [95% UB = 27.2 to 28.1] years of age) but is lower than the expected life expectancy (82.2 years of age) of an HIV-negative male in the general population. The gap between life expectancy among PLHIV and the general population is greater for those infected at younger ages.

**Figure:**
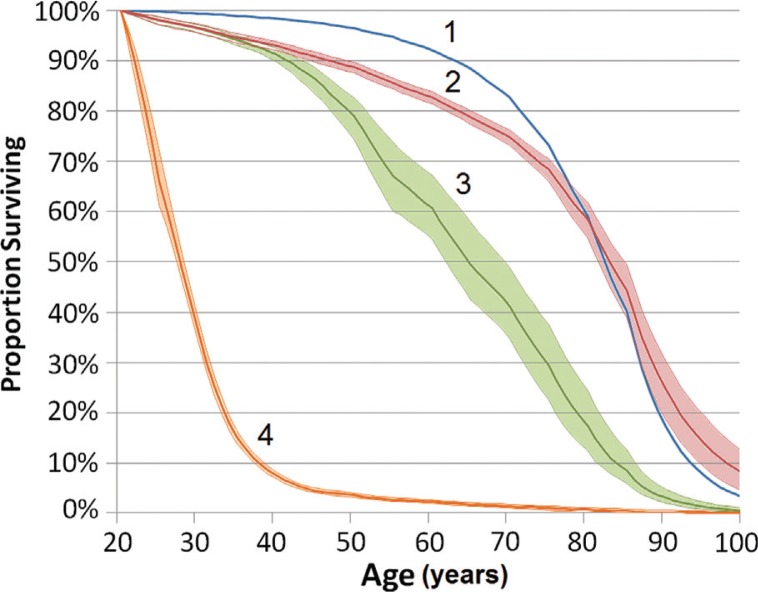

**Conclusions:**

As treatment options are exhausted in the coming years, a substantial difference in life expectancy between PLHIV and the general population is expected, particularly for people who acquire HIV at a younger age or who are currently highly treatment-experienced.

